# Interfractional Variations of Tumor Centroid Position and Tumor Regression during Stereotactic Body Radiotherapy for Lung Tumor

**DOI:** 10.1155/2014/372738

**Published:** 2014-12-07

**Authors:** Yanan Sun, Yufei Lu, Siguo Cheng, Wei Guo, Ke Ye, Huiyun Zhao, Xiaoli Zheng, Dingjie Li, Shujuan Wang, Chengliang Yang, Hong Ge

**Affiliations:** ^1^Department of Radiation Oncology, The Affiliated Cancer Hospital of Zhengzhou University, 127 Dongming Road, Zhengzhou, Henan 450008, China; ^2^Red Cross Blood Center of Henan, 9 Tongle Road, Zhengzhou, Henan 450053, China

## Abstract

*Purpose*. To determine interfractional changes of lung tumor centroid position and tumor regression during stereotactic body radiation therapy (SBRT). *Methods and Materials*. 34 patients were treated by SBRT in 4-5 fractions to a median dose of 50 Gy. The CT scans acquired for verification were registered with simulation CT scans. The gross target volume (GTV) was contoured on all verification CT scans and compared to the initial GTV in treatment plan system. *Results*. The mean (±standard deviation, SD) three-dimension vector shift was 5.2 ± 3.1 mm. The mean (±SD) interfractional variations of tumor centroid position were −0.7 ± 4.5 mm in anterior-posterior (AP) direction, 0.2 ± 3.1 mm in superior-inferior (SI) direction, and 0.4 ± 2.4 mm in right-left (RL) direction. Large interfractional variations (≥5 mm) were observed in 5 fractions (3.3%) in RL direction, 16 fractions (10.5%) in SI direction, and 36 fractions (23.5%) in AP direction. Tumor volume did not decrease significantly during lung SBRT. *Conclusions*. Small but insignificant tumor volume regression was observed during lung SBRT. While the mean interfractional variations of tumor centroid position were minimal in three directions, variations more than 5 mm account for approximately a third of all, indicating additional margin for PTV, especially in AP direction.

## 1. Introduction

Stereotactic body radiation therapy (SBRT) is an emerging radiotherapy technique, characterized by delivering high doses in a few fractions (typically between 1 and 5 fractions) [1]. Consequently, this hypofractionated radiotherapy technique results in a high biologically equivalent dose (BED) compared with conventional radiotherapy treatment. On one hand, SBRT with a BED more than 100 Gy is deemed as effective in improving local control rate for early stage lung cancer [2]; on the other hand, the toxicity of critical nearby normal tissue also is dose-dependent. In order to limit the risk of treatment toxicity, the volume of normal tissue receiving high doses outside the target should be minimized, which indicates that the gradient describing the dose fall-off outside the target should be sharp [3]. This perfect state can only be achieved when the target is absolutely motionless. Yet, such an extent is almost impossible for moving organs such as lung and liver. Judging from fluoroscopy, tumor motion will result in a certain degree of tumor volume missing and mistaken radiation delivered to normal tissues [4]. This phenomenon is especially obvious in lung tumors located in the lower lobe. Various methods have been developed for tumor motion management: restricting diaphragm motion with the use of abdominal compression or breath hold techniques [5, 6]; generating an internal target volume (ITV) including tumor motion trajectory at quiet respiration with the use of four-dimension computed tomograph (4D-CT) [7]; and delivering respiratory gated radiotherapy with the use of tracking technique [8].

Even if tumor motion could be well-managed using the above-mentioned methods, an underlying assumption of radiation delivery to lung tumor is that patient's respiratory pattern is consistent during treatment. However, intrafractional and interfractional variations of tumor position during treatment may compromise the accuracy of radiation delivery [9, 10]. Furthermore, variation of tumor volume in response to radiation treatment may impact accurate delivery. During conventional radiotherapy treatment for lung cancer, though a notable heterogeneity of tumor volume change was observed, a trend of decrease in average tumor volume was also detected after full course of treatment [9, 11–13]. Unlike conventional radiotherapy, SBRT involves high fractionated doses delivered within short time, leading to possible different patterns of variation in tumor position and volume. The aim of this study was to evaluate the interfractional variations of tumor centroid position and GTV changes over time during SBRT for primary or metastatic lung tumor.

## 2. Methods and Materials

### 2.1. Patients Screening

From October 2011 to March 2014, a total of 38 patients with primary or metastatic lung tumors underwent SBRT treatment in our institution. However, 4 patients did not complete the full course. Finally, relatively integrated data were available in 34 patients. Medical records and CT images of these patients were collected and analyzed. 23 patients had peripheral tumors and 11 patients had central lung tumors. Of these patients, positron emission tomography (PET)/CT was used for lung tumor diagnosis in 7 patients and CT was used in 27 patients. Adopted radiotherapy regimens were summarized as follows: 50 Gy in 5 fractions for 16 patients; 48 Gy in 4 fractions for 10 patients; 55 Gy in 5 fractions for 3 patients; 40 Gy in 4 fractions for 3 patients; 45 Gy in 5 fractions for 1 patient; and 50 Gy in 4 fractions for 1 patient. This study was approved by the institutional review board and the patient informed consent was written by each patient or patient's relative before SBRT. Details of baseline characteristics of patients and tumors are showed in Table 1.

### 2.2. Treatment Planning

Patients were immobilized head-first-supine without abdominal compression in an evacuated immobilization bag (Calergo). All patients were instructed to lie down in their neutral positions with arms above head. Prior to simulation, lung tumor motion amplitude was assessed on fluoroscopy. Peak-to-peak motion amplitude of lung tumor was measured based on the center of lung tumor other than the edge of tumor volume. Treatment simulation was conducted on a 16-slice CT scanner (Brilliance CT; Big Bore; Philips). Relevant parameters were as follows: 120 kV; 350 mAs; pitch: 0.938; slice thickness: 3 millimeters. Patients were instructed to breathe quietly during scanning and the free-breathing helical CT scanning was carried out ranging from the lower neck to base of lung to encompass the entire thoracic region. Each CT scanning took approximately 13 seconds. Then, acquired CT image data were imported to the Prowess Panther 5.10 treatment planning system (Siemens Medical System).

For consistent evaluation of tumor volume, only visible tumor in CT image was considered as GTV. Contours of GTV were delineated on axial images. When tumors were located at pulmonary parenchyma away from soft tissues of mediastinum or chest wall, GTV contours were drawn using lung window (window width 1000/window location −650). When lung tumor is abutted to mediastinum or chest wall, mediastinum window (window width 350/window location 40) was also employed to distinguish the border of GTV from soft tissues. PTV was generated by adding an isotropic margin of 5 mm to the GTV in both anterior-posterior (AP) direction and right-left direction and 10 mm to the GTV in superior-inferior (SI) direction. The treatment plan was designed to deliver the prescription dose to 95% of the PTV. To ensure the consistency of GTV contouring and interfractional tumor position assessment, all contours were completed by a single radiation oncologist, under supervision of two other senior radiation oncologists with lung SBRT expertise.

### 2.3. Verification of Set-Up

The time interval between simulation and the first fraction of radiotherapy ranged from 1 to 11 days with a mean of 3.6 days. All patients were treated on an integrated CT-LINIC system (CT vision, Siemens). Before each fraction of radiation delivery, a CT scan was performed for the patient. In order to reduce imaging radiation dose to the patients, these verification CT scans covered only the region from 5 cm above the superior border of tumor to 5 cm below the inferior border of tumor. The scanning time ranged from 5 to 8 seconds, which was shorter than that of the initial simulation CT scan. Then, the acquired CT scan was imported into an in-house developed image registration program. The set-up procedure was performed according to the location of lung tumors. For the part of lung tumors located near thoracic vertebra, the set-up error was calculated based on bony anatomy. For the part of lung tumors located away from thoracic vertebra, the set-up error was calculated based on soft tissue. Set-up errors were calculated in three dimensions: superior-inferior (SI), anterior-posterior (AP), and right-left (RL). If set-up error was more than 2 mm in any direction, the patient would be repositioned and given another CT scan. Not until set-up error was less than or equal to 1 mm could the radiotherapy treatment begin. The full course of radiotherapy treatment lasted from 7 to 14 days with a mean of 10 days.

### 2.4. Data Analysis of Interfractional Variation in Tumor Volume and Position

Each treatment course consisted of 4 or 5 SBRT fractions. Though a total of 20 patients underwent the fifth fraction of SBRT, the CT scans were available in only 17 cases. All patients had in-room CT scanning taken prior to each fraction of SBRT for verification. Thus, a total of 153 verification CT scans were acquired. All of them were registered with their corresponding reference CT scans. The registration procedure was manually performed on the basis of the bony reference region of interest (ROI) defined on reference CT, in which the vertebrae adjacent to tumor in the treatment planning image were used as reference. Translational shifts were performed in SI, AP, and RL directions and rotation shifts were adjusted to make sure of the best match. Then, contours of gross tumor in 4 or 5 fractions were delineated for each patient according to the method mentioned in treatment planning. In order to eliminate subjective bias, the physician was blind to the date of CT images he delineated when performing this procedure.

Using Prowess Panther 5.10 software, the *X*-lateral (RL), *Y*-lateral (SI), and *Z*-lateral (AP) directions of the center of GTVs derived from initial treatment planning and each SBRT fraction were established. Interfractional variations in GTV were obtained through comparing the center of GTV of each verification CT scan with that of the coregistered planning CT scan. Then, the 3D vector shift magnitudes were also calculated by taking the root sum squares of interfractional variations in three directions.

### 2.5. Statistical Analysis

Generalized estimating equation for repeated measures data was adopted to examining the time trend in mean GTVs and 3D vector shift during the course of SBRT. In this equation, both the size and location of tumor were set as predictive factors to explore their relationship with interfractional change of GTV. Because variables did not conform to the assumption of normality or homogeneity of variance, rank-sum test such as Kruskal-Wallis *H* test was used to compare 3D vector shift in different parts of lung and in different sizes of tumors. Comparison of the planning GTVs and GTVs obtained from the first verification CT scans adopted paired *t*-test. Spearman rank correlation was used to determine the relationship between the 3D shift vector of tumor motion and interfractional tumor centroid position. All statistical procedures were carried out in SPSS Statistic 22.0.

## 3. Results

### 3.1. Tumor Motion Assessed on Fluoroscopy before Simulation

The overall mean ± SD of tumor motion amplitude was 9.7 ± 2.7 mm (range: 5–15 mm) in the SI direction, 3.4 ± 0.9 mm (range: 2–5 mm) in the AP direction, and 2.7 ± 0.8 mm (range: 1–4 mm) in the RL direction. When patients were stratified by tumor location, the mean ± SD of motion amplitudes is detailed in Table 2.

### 3.2. Interfractional Variation of Tumor Centroid Position

**Figure 1:**
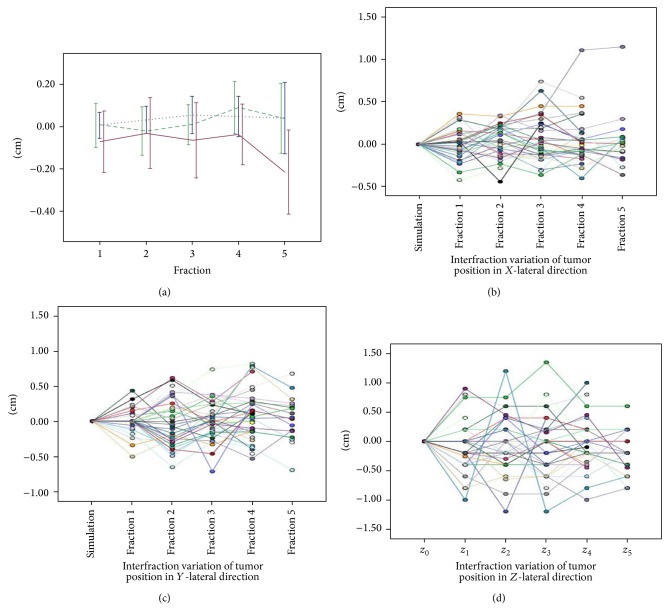
(a) shows the mean interfraction variation of tumor centroid position over time. The blue, green, and red lines represent the interfraction variation of tumor centroid position in RL, AP, and SI directions, respectively. Horizontal lines represent the mean interfraction variation of tumor centroid position. Vertical lines represent the 95% error bars. (b), (c), and (d) show interfraction variations of tumor position for each patient during treatment in *X*-lateral (right-left), *Y*-lateral (superior-inferior), and *Z*-lateral (anterior-posterior) directions. Each color represents the interfraction variation of tumor position of one patient.

Figure 1(a) shows the mean interfractional variation of tumor centroid position over time. Separate curves for each patient are shown in Figures 1(b), 1(c), and 1(d). Although overall variations were minimal in all directions, large deviations were seen in the AP direction, with a mean ± SD of −0.7 ± 4.5 mm, as compared with 0.2 ± 3.1 mm in SI direction and 0.4 ± 2.4 mm in RL direction. Of all 153 fractions, absolute interfractional variations that were greater than 5 mm were observed in 5 fractions (3.3%) in the RL direction, 16 fractions (10.5%) in SI direction, and 36 fractions (23.5%) in the AP direction. When setting ≥7 mm as threshold, the corresponding values were 3 fractions (2.0%) in RL direction, 7 fractions (4.6%) in SI direction, and 20 fractions (13.1%) in AP direction. When setting ≥10 mm as threshold, the corresponding values were 2 fractions (1.3%) in RL direction, 0 fraction (0%) in SI direction, and 7 fractions (4.6%) in AP direction. According to a recent research performed by Zhang et al. [14], the one dimension asymmetric expansion for single fraction is *C*_*i*_ = 2.33^*^(SD), so we could deduce that the GTV-PTV margins for AP, SI, and RL directions were 10.4 mm, 7.3 mm, and 5.5 mm, respectively. The mean magnitude of interfractional variations of GTV centroid position classified by the location of tumor is summarized in Table 3.

**Figure 2:**
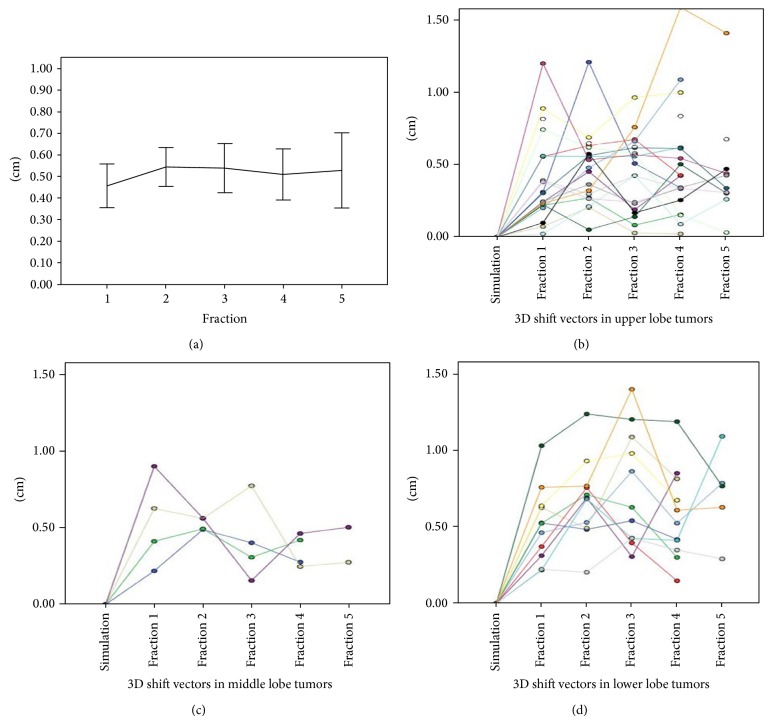
(a) shows the mean 3D shift of tumor centroid position over time. Horizontal lines represent the mean 3D shift of tumor centroid position. Vertical lines represent the 95% error bars. (b), (c), and (d) show the 3D vector shift vectors in upper, middle, and lower lobe tumors for each patient. Each color represents the interfractional variation of tumor position of one patient.

Figure 2(a) shows 3D vector shift of interfractional tumor centroid position over time. Correlation analysis showed that the mean 3D vector shift of interfractional tumor centroid position was not related to that of tumor motion (*r* = 0.292, *P* = 0.093). The overall 3D vector shift was calculated from all fractions, with a mean ± SD of 5.2 ± 3.1 mm. No trend was observed from the beginning to the end of SBRT treatment (*P* = 0.452). If the location of tumor was used as stratification factor, greater tumor motion was observed in lower lobe tumors with a mean ± SD of 6.4 ± 3.0 mm, as compared with 4.6 ± 3.1 mm (*P* = 0.001) in upper lobe tumors and 4.5 ± 1.9 mm (*P* = 0.020) in middle lobe tumors. Figures 2(b), 2(c), and 2(d) show the magnitude of 3D vector shift classified by tumor location. Tumor size did not seem to impact the 3D vector shift (*P* = 0.248).

### 3.3. Change of GTV during SBRT Treatment

Comparison between treatment planning GTVs and GTVs contoured from the first verification CT scans did not show statistical significance (*P* = 0.569). To eliminate clinical effect on tumors caused by other treatments such as chemotherapy and biologic treatment, GTVs obtained from the first verification CT scans were used as the references to assess GTV change during SBRT. The result of generalized estimating equation for repeated measures showed that GTVs did not decrease significantly along with the elapse of time from the beginning to the end of SBRT treatment (*P* = 0.078). Lung tumors located in different lobes did not show significant difference (*P* > 0.05), but tumors with a size ≥3 cm showed larger GTV decrease compared with those <3 cm (*P* < 0.001).

**Figure 3:**
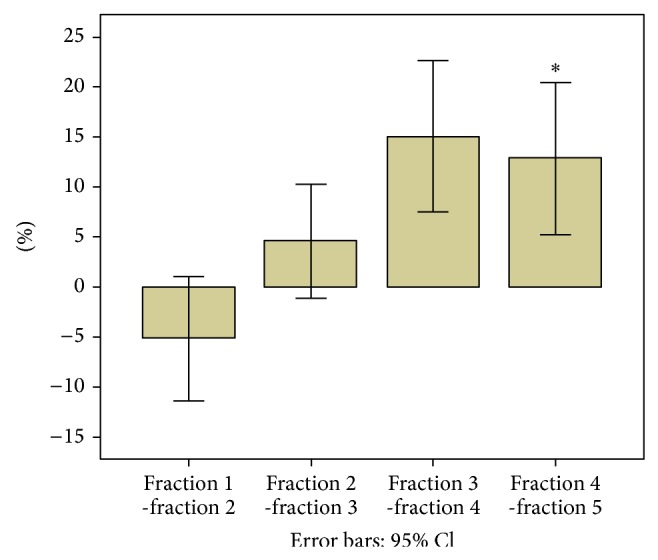
It shows the percentage GTV reduction over time during SBRT treatment. Bars represent the percentage GTV reduction in each fraction. The reference GTV is that obtained from the first verification CT. Vertical lines represent the 95% error bars. ^*^Because only 17 verification CT scans were available in the fifth fraction of SBRT, the fourth bar represents only half patients.

The percentile GTV reduction during SBRT is shown in Figure 3. Because only 17 verification CT scans were available in the fifth fraction of SBRT, the last bar only represented the percentile GTV reduction of half patients. As shown in the table, the mean of GTVs increased by approximate 5% before the second fraction of SBRT transiently. The increase of GTV was observed in 15 tumors, of which 8 tumors showed a ≥20% increase and 4 tumors showed a ≥30% increase. From then on, the decrease of GTV was consistent. Although the mean of overall percentile GTV deduction was 12.8% before the final fraction of SBRT, the mean absolute value of GTV decrease was 1.53 cm^3^ per tumor.

## 4. Discussion

In this study, all patients were treated using an integrated CT-LINAC system and CT scans were performed before each SBRT fraction. We found that tumor volume did not decrease significantly during SBRT treatment (*P* = 0.078). The mean of overall percentile GTV reduction was 12.8% and the mean absolute GTV reduction was 1.53 cm^3^. A few studies reported similar results that no obvious tumor volume reduction was observed immediately after SBRT treatment [15–17]. Several reasons may contribute to this phenomenon: firstly, because the maximum diameter of each tumor was less than 5 cm, the absolute value of GTV was small correspondingly; secondly, tumor regression is a complex radiobiological phenomenon during fractionated radiotherapy, incorporating combined influence of cell loss, changing tumor kinetics, repair, reoxygenation, and clearance of the dead and necrotic tissue/debris; thirdly, time factor also plays an important role in GTV change during SBRT. SBRT delivers an effective radiation dose with high accuracy in a hypofractionated fashion, typically in 3 to 5 fractions within two weeks. However, significant tumor volume reduction occurred after 4 weeks of SBRT treatment [18]. Other studies even reveal that the tumor regression and reduction of glucose metabolism can last for 24 months [19, 20]. In addition, we found a transient increase in the mean percentile GTV reduction before the second fraction of SBRT. A possible reason is that a single high dose delivered in short time might induce reactive edema and inflammation of tumor cells, which would lead to a slight increase of tumor volume. Thus, image guidance during SBRT treatment is prerequisite and additional margin should be considered in consideration of this fact.

Though the mean 3D vector shift of interfractional tumor centroid position was not closely related to that of tumor motion, there were many other factors, such as variation of respiratory frequency and amplitude, heartbeat, complication during treatment, tumor volume change, and scanning speed of 3DCT. Tumor motion amplitude assessed with fluoroscopy just once before treatment could not predict the ever-changing situation during SBRT treatment. Our results demonstrate that large deviation of tumor centroid position was in AP direction after registration with 3D simulation CT scans, with a mean ± SD of −0.7 ± 4.5 mm, as compared with 0.2 ± 3.1 mm in SI direction and 0.4 ± 2.4 mm in RL direction. Absolute interfractional variations ≥5 mm (any direction) were observed in 48 fractions (31.4%), primarily in AP direction. In this study, the contour of GTV was based on the image series of three-dimension computed tomography (3DCT). The margin added to GTV and the contour of PTV referred to the standard of RTOG 0915: an isotropic margin of 5 mm to the GTV in both AP direction and RL direction and 10 mm to the GTV in SI direction. According to our result, it seems to be insufficient to add a margin of 5 mm in AP direction and 10 mm might be appropriate based on the formula of Zhang et al. [14]. Great interfractional variation of tumor motion was observed in lower lobe tumors for larger mean 3D vector shift compared with upper or middle lobe tumors. The change of frequency and magnitude of respiratory and heartbeat may contribute to interfractional variation of tumor centroid position [21]. In addition, because the most part of patients (85.3%) involved in this study were diagnosed as advanced stage lung tumor or metastatic lung tumor, their physiological condition was complicated and susceptible to complications. Mild pneumothorax (1 case), pleural diffusion (2 cases), and pneumonitis (4 cases) developed during SBRT treatment. In a similar study performed by M.D. Anderson Cancer Center [22], both the free-breathing helical CT scan and the 4DCT scan were used for SBRT treatment simulation, and daily GTV deviations relative to bony references in a total of 117 tumors were evaluated. Eventually, an almost Gaussian distribution was obtained with a mean close to zero in three directions. For the 76 cases in which a free-breathing CT scan was used as the reference, the mean (±SD) daily GTV deviation from the bone position was 0.3 ± 3.8 mm in the AP direction, 0.4 ± 4.0 mm in the SI direction, and 0.4 ± 2.6 mm in the RL direction. For all patients, a clinically remarkable trend (net change >5 mm in any direction) in GTV centroid position was observed in 23 cases (20%). Bissonnette et al. [17] evaluated intrafractional and interfractional change in tumor motion amplitude over a SBRT course delivered in three fractions. Both 4DCT planning scan and free-breathing helical scan were used for simulation in 18 patients. Respiratory correlated cone beam computed tomography (rcCBCT) was performed at the beginning, midpoint, and end of each fraction of SBRT. Observed intrafraction change in tumor motion was, on average, <1 mm and none of them was statistically different with respect to the reference rcCBCT. Interfractional changes in tumor motion were 0.4, 1.0, and 0.4 mm in the RL, SI, and AP directions compared with the motion recorded on 4DCT. Though different scanning models were adopted to perform simulation or verification, similar interfractional variation of tumor centroid position with a mean close to zero was obtained, which indicates the reliability of CT-on rail system being used for verification.

Computed tomography on-rail (CT on-rail) system has been used to guide SBRT as an in-room computed tomography method [22]. Compared with electronic portal imaging (EPID), it has the advantage of calibrating set-up error in three dimensions with high resolution and accuracy. Furthermore, it is consistent with the imagery method of simulation CT scanning, which makes direct comparison among GTVs achievable during treatment. However, because the scanning speed of CT on-rail system is much faster than 4DCT or cone beam computed tomography (CBCT), the tumor is in an arbitrary position when undergoing scanning. Meanwhile, 3DCT scans also took time in image acquisition for the tumor volume, so simulation and verification CT images also cover a certain degree of tumor motion information, which could cause the uncertainty in measurement of tumor centroid position. However, according to our previous study [7], even the 4DCT was suboptimal to determine the ITV. The combination of 4DCT and free breathing 3DCT can potentially minimize the uncertainty of tumor volume. Besides, even if just a single physician was required to perform the registration procedure and contour GTV to guarantee the consistency in evaluation, there still exist many uncertain factors, such as motion artifacts induced by fast helical CT and pneumonia developed during treatment. All these factors would affect the accuracy of GTV contour and further the evaluation of interfractional variation of tumor centroid position. Furthermore, though no significant decrease of GTV was observed during SBRT treatment, there was a trend towards decrease and further follow-up is needed to evaluate the critical time.

## 5. Conclusion

Small but insignificant tumor volume regression was observed during lung SBRT. It should be not necessary to perform another field shrink during SBRT treatment as conventional radiotherapy. However, a transient increase in GTV occurred after the first fraction of SBRT and thereafter the trend of decrease was persistent, so the margin added to GTV should consider these variations. While the mean interfractional variations of tumor centroid position were minimal in three directions, variations more than 5 mm account for approximately a third of all, indicating additional margin for PTV, especially in AP direction. Ideally, individualized PTV for lung SBRT should be determined by combination of interfractional variations of tumor centroid position and tumor regression as well as daily set-up error. Regardless, the final check should be performed with image-guidance for the verification of treatment target volume to prevent target from underdosing and normal tissue from overdosing.

## Figures and Tables

**Table 1:** Baseline characteristics of patients and tumors.

Factors	Number of patients	Patient (%)
Gender		
Male	21	61.8
Female	13	38.2
Age (year)		
<60	18	52.9
≥60	16	47.1
Lung tumor		
Primary lung tumor		47.1
I A	1	2.9
I B	4	11.8
IV	11	32.4
^*^Recurrence of lung tumor	4	11.8
Metastatic lung tumor	14	41.2
Tumor size (cm)		
<3.0	19	55.9
≥3.0 and ≤5.0	15	44.1
Primary site of tumor		
Lung	20	58.8
Colon	4	11.8
Esophagus	4	11.8
Head and neck	3	8.7
Breast	1	2.9
Cervix	1	2.9
Thymus	1	2.9
Histologic classification		
Squamous cell carcinoma	14	41.2
Adenocarcinoma	13	38.2
Small cell carcinoma	1	2.9
Adenoid cystic carcinoma	1	2.9
NOS	5	14.7
Type of lung tumor		
Central	11	32.4
Peripheral	23	67.6
Location of lung tumor		
Left upper lobe	12	35.3
Left lower lobe	7	20.6
Right upper lobe	7	20.6
Right middle lobe	4	11.8
Right lower lobe	4	11.8
Previous treatment		
Chemotherapy	7	20.6
Conventional radiotherapy	5	14.7
No	22	64.7

NOS = not otherwise specified.

^*^Recurrence of lung tumor means that lung tumor relapses after complete surgical resection.

**Table 2:** Magnitude (in millimeters) of tumor motion assessed on fluoroscopy.

Location of lung tumor	SI (Mean ± SD)	AP (Mean ± SD)	RL (Mean ± SD)
Upper lobe	8.4 ± 2.3	3.0 ± 0.8	2.5 ± 0.7
Middle lobe	10.8 ± 9.6	3.6 ± 0.5	3.0 ± 0.8
Lower lobe	11.6 ± 2.6	3.8 ± 1.2	3.0 ± 0.8

Total	9.7 ± 2.7	3.4 ± 0.9	2.7 ± 0.8

SI = superior-inferior; AP = anterior-posterior; RL = right-left; SD = standard deviation.

**Table 3:** Interfraction variation (in millimeters) of GTV centroid position classified by the location of lung tumor.

Location	SI (Mean ± SD)	AP (Mean ± SD)	RL (Mean ± SD)
Upper lobe	−0.29 ± 2.77	−0.50 ± 3.90	0.88 ± 2.62
Middle lobe	−0.76 ± 2.76	1.17 ± 3.72	−0.01 ± 1.30
Lower lobe	−1.71 ± 5.38	1.52 ± 3.49	−0.43 ± 1.93

Total	0.23 ± 3.13	−0.69 ± 4.47	0.36 ± 2.36

SI = superior-inferior; AP = anterior-posterior; RL = right-left; SD = standard deviation.

## References

[1] Potters L., Kavanagh B., Galvin J. M., Hevezi J. M., Janjan N. A., Larson D. A., Mehta M. P., Ryu S., Steinberg M., Timmerman R., Welsh J. S., Rosenthal S. A. (2010). American Society for Therapeutic Radiology and Oncology (ASTRO) and American College of Radiology (ACR) practice guideline for the performance of stereotactic body radiation therapy. *International Journal of Radiation Oncology Biology Physics*.

[2] Onishi H., Araki T., Shirato H., Nagata Y., Hiraoka M., Gomi K., Yamashita T., Niibe Y., Karasawa K., Hayakawa K., Takai Y., Kimura T., Hirokawa Y., Takeda A., Ouchi A., Hareyama M., Kokubo M., Hara R., Itami J., Yamada K. (2004). Stereotactic hypofractionated high-dose irradiation for stage I nonsmall cell lung carcinoma: Clinical outcomes in 245 subjects in a Japanese multiinstitutional study. *Cancer*.

[3] Benedict S. H., Yenice K. M., Followill D., Galvin J. M., Hinson W., Kavanagh B., Keall P., Lovelock M., Meeks S., Papiez L., Purdie T., Sadagopan R., Schell M. C., Salter B., Schlesinger D. J., Shiu A. S., Solberg T., Song D. Y., Stieber V., Timmerman R., Tomé W. A., Verellen D., Wang L., Yin F.-F. (2010). Stereotactic body radiation therapy: the report of AAPM Task Group 101. *Medical Physics*.

[4] Sixel K. E., Ruschin M., Tirona R., Cheung P. C. F. (2003). Digital fluoroscopy to quantify lung tumor motion: potential for patient-specific planning target volumes. *International Journal of Radiation Oncology Biology Physics*.

[5] Bouilhol G., Ayadi M., Rit S., Thengumpallil S., Schaerer J., Vandemeulebroucke J., Claude L., Sarrut D. (2013). Is abdominal compression useful in lung stereotactic body radiation therapy? A 4DCT and dosimetric lobe-dependent study. *Physica Medica*.

[6] Peng Y., Vedam S., Chang J. Y., Gao S., Sadagopan R., Bues M., Balter P. (2011). Implementation of feedback-guided voluntary breath-hold gating for cone beam CT-based stereotactic body radiotherapy. *International Journal of Radiation Oncology, Biology, Physics*.

[7] Ge H., Cai J., Kelsey C. R., Yin F.-F. (2013). Quantification and minimization of uncertainties of internal target volume for stereotactic body radiation therapy of lung cancer. *International Journal of Radiation Oncology Biology Physics*.

[8] Guckenberger M., Krieger T., Richter A., Baier K., Wilbert J., Sweeney R. A., Flentje M. (2009). Potential of image-guidance, gating and real-time tracking to improve accuracy in pulmonary stereotactic body radiotherapy. *Radiotherapy and Oncology*.

[9] Britton K. R., Starkschall G., Tucker S. L., Pan T., Nelson C., Chang J. Y., Cox J. D., Mohan R., Komaki R. (2007). Assessment of gross tumor volume regression and motion changes during radiotherapy for non-small cell lung cancer as measured by four-dimensional computed tomography. *International Journal of Radiation Oncology Biology Physics*.

[10] Bissonnette J. P., Franks K. N., Purdie T. G., Moseley D. J., Sonke J. J., Jaffray D. A., Dawson L. A., Bezjak A. (2009). Quantifying interfraction and intrafraction tumor motion in lung stereotactic body radiotherapy using respiration-correlated cone-beam computed tomography. *International Journal of Radiation Oncology Biology Physics*.

[11] Juhler-Nøttrup T., Korreman S. S., Pedersen A. N., Persson G. F., Aarup L. R., Nyström H., Olsen M., Tarnavski N., Specht L. (2008). Interfractional changes in tumour volume and position during entire radiotherapy courses for lung cancer with respiratory gating and image guidance. *Acta Oncologica*.

[12] Siker M. L., Tomé W. A., Mehta M. P. (2006). Tumor volume changes on serial imaging with megavoltage CT for non-small-cell lung cancer during intensity-modulated radiotherapy: how reliable, consistent, and meaningful is the effect?. *International Journal of Radiation Oncology Biology Physics*.

[13] Bosmans G., van Baardwijk A., Dekker A., Öllers M., Boersma L., Minken A., Lambin P., De Ruysscher D. (2006). Intra-patient variability of tumor volume and tumor motion during conventionally fractionated radiotherapy for locally advanced non-small-cell lung cancer: a prospective clinical study. *International Journal of Radiation Oncology Biology Physics*.

[14] Zhang Q., Chan M. F., Burman C., Song Y., Zhang M. (2013). Three independent one-dimensional margins for single-fraction frameless stereotactic radiosurgery brain cases using CBCT. *Medical Physics*.

[15] Matsugi K., Narita Y., Sawada A., Nakamura M., Miyabe Y., Matsuo Y., Narabayashi M., Norihisa Y., Mizowaki T., Hiraoka M. (2009). Measurement of interfraction variations in position and size of target volumes in stereotactic body radiotherapy for lung cancer. *International Journal of Radiation Oncology Biology Physics*.

[16] Hodge W., Tomé W. A., Jaradat H. A., Orton N. P., Khuntia D., Traynor A., Weigel T., Mehta M. P. (2006). Feasibility report of image guided stereotactic body radiotherapy (IG-SBRT) with tomotherapy for early stage medically inoperable lung cancer using extreme hypofractionation. *Acta Oncologica*.

[17] Bissonnette J.-P., Franks K. N., Purdie T. G., Moseley D. J., Sonke J.-J., Jaffray D. A., Dawson L. A., Bezjak A. (2009). Quantifying interfraction and intrafraction tumor motion in lung stereotactic body radiotherapy using respiration-correlated cone-beam computed tomography. *International Journal of Radiation Oncology Biology Physics*.

[18] Underberg R. W., Lagerwaard F. J., Van Tinteren H., Cuijpers J. P., Slotman B. J., Senan S. (2006). Time trends in target volumes for stage I non-small-cell lung cancer after stereotactic radiotherapy. *International Journal of Radiation Oncology Biology Physics*.

[19] Dahele M., Palma D., Lagerwaard F., Slotman B., Senan S. (2011). Radiological changes after stereotactic radiotherapy for stage I lung cancer. *Journal of Thoracic Oncology*.

[20] Matsuo Y., Nakamoto Y., Nagata Y., Shibuya K., Takayama K., Norihisa Y., Narabayashi M., Mizowaki T., Saga T., Higashi T., Togashi K., Hiraoka M. (2010). Characterization of FDG-PET images after stereotactic body radiation therapy for lung cancer. *Radiotherapy and Oncology*.

[21] Seppenwoolde Y., Shirato H., Kitamura K., Shimizu S., Van Herk M., Lebesque J. V., Miyasaka K. (2002). Precise and real-time measurement of 3D tumor motion in lung due to breathing and heartbeat, measured during radiotherapy. *International Journal of Radiation Oncology Biology Physics*.

[22] Ikushima H., Balter P., Komaki R., Hunjun S., Bucci M. K., Liao Z., McAleer M. F., Yu Z. H., Zhang Y., Chang J. Y., Dong L. (2011). Daily alignment results of in-room computed tomography-guided stereotactic body radiation therapy for lung cancer. *International Journal of Radiation Oncology Biology Physics*.

